# Opioids in Post-stroke Pain: A Systematic Review and Meta-Analysis

**DOI:** 10.3389/fphar.2020.587050

**Published:** 2020-11-27

**Authors:** Damiana Scuteri, Elisa Mantovani, Stefano Tamburin, Giorgio Sandrini, Maria Tiziana Corasaniti, Giacinto Bagetta, Paolo Tonin

**Affiliations:** ^1^ Pharmacotechnology Documentation and Transfer Unit, Section of Preclinical and Translational Pharmacology, Department of Pharmacy, Health and Nutritional Sciences, University of Calabria, Rende, Italy; ^2^ Department of Neurosciences, Biomedicine and Movement Sciences, University of Verona, Verona, Italy; ^3^ Department of Brain and Behavioral Sciences, University of Pavia, IRCCS C. Mondino Foundation Neurologic Institute, Pavia, Italy; ^4^ Department of Health Sciences, University “Magna Graecia” of Catanzaro, Catanzaro, Italy; ^5^ School of Hospital Pharmacy, University "Magna Graecia" of Catanzaro, Catanzaro, Italy; ^6^ Regional Center for Serious Brain Injuries, S. Anna Institute, Crotone, Italy

**Keywords:** post-stroke pain, stroke, pain, central pain, opioids, rehabilitation, systematic review, meta-analysis

## Abstract

**Background:** Post-stroke pain is one of the most common *sequelae* of stroke, which stands among the leading causes of death and adult-acquired disability worldwide. The role and clinical efficacy of opioids in post-stroke pain syndromes is still debated.

**Objectives:** Due to the important gap in knowledge on the management of post-stroke pain, this systematic review aimed at assessing the efficacy of opioids in post-stroke pain syndromes.

**Methods:** A literature search was conducted on databases relevant for medical scientific literature, i.e. PubMed/MEDLINE, Scopus, Web of Science and Cochrane Library databases from databases inception until August 31^st^, 2020 for clinical trials assessing the effects of opioids and opioid antagonists on pain reduction and pain related symptoms in patients with post-stroke pain syndromes. Studies assessing the effects of other medications (e.g., tricyclic antidepressant, pregabalin) or non - pharmacological management strategies (e.g., neurostimulation techniques) were excluded. The selected studies have been subjected to examination of the risk of bias.

**Results:** The literature search retrieved 83,435 results. After duplicates removal, 34,285 articles were title and abstract screened. 25 full texts were assessed and 8 articles were identified to be eligible for inclusion in the qualitative summary and narrative analysis, of which three were placebo-controlled and two were dose-response. Among placebo-controlled studies, two evaluated the analgesic effect of morphine and one assessed the effects of the opioid antagonist naloxone on patients with central post-stroke pain. With regard to dose-response studies, both were on patients with central post-stroke pain, one assessing the efficacy of levorphanol, and the other on naloxone. Seven out of eight included studies showed an overall slight analgesic effect of opioids, with less consistent effects on other pain-related symptoms (e.g., mood, quality of life). The randomized controlled trials were subjected to meta-analysis and rating of the quality of evidence for the two outcomes considered according to GRADE (Grading of Recommendations, Assessment, Development and Evaluations) system. The overall results are inconclusive because of the small number of studies and of patients.

**Conclusions:** The limited number of the included studies and their heterogeneity in terms of study design do not support the efficacy of opioids in post-stroke pain and in pain-related outcomes. Large double-blind randomized clinical trials with objective assessment of pain and related symptoms are needed to further investigate this topic.

## Introduction

### Post-stroke Pain

Stroke stands among the leading causes of death and adult-acquired disability with 13.7 million new strokes every year worldwide (Collaborators, 2019). Post-stroke pain is one of the most poorly understood complications, arising either in the acute, but mainly in the subacute or chronic stages (i.e., often within 6 months) of stroke (Merskey, 1994) . The prevalence of post-stroke pain varies largely depending on the definition of pain; the musculoskeletal pain appears to be the most common being reported in up to 72% of stroke patients (Harrison and Field, 2015). While post-stroke pain syndromes in general are estimated to affect up to 30–40% of stroke survivors (Paolucci et al., 2016), central post-stroke pain (CPSP) is more rare: definite CPSP was found in 3.5%, definite/probable in 5.8% and CPSP-like pain or dysesthesia in 6.7% of patients in a specific population-based study of post-stroke pain (Klit et al., 2011). Pain after stroke can remarkably reduce the quality of life, causing depression, anxiety and sleep disorders making rehabilitation more difficult.

### Post-stroke Pain Syndromes

Pain after stroke is often under-reported, being diagnosed only if actively searched by the clinician (Harrison and Field, 2015). There are multiple types of post-stroke pain syndromes that can also occur in combination, with both neuropathic and nociceptive features. The most common types of pain after stroke include CPSP, pain secondary to spasticity, shoulder pain, complex regional pain syndrome (i.e., CRPS), and headache (O’Donnell et al., 2013). Dysesthesia and allodynia often occur and the symptoms generally develop within the area corresponding to the lesion with frequent involvement of face, hand and foot, but sometimes also of thigh and shoulder (Kim, 2014). In particular, CPSP is often characterized by dysesthesia, constant or intermittent pain and hyperalgesia/allodynia (Harrison and Field, 2015). CPRS is of type I when nerve lesion is not identifiable, while of type II when there is a definite nerve lesion.

### Treatment and Limitations

Treatment of post-stroke pain is made challenging by the lack of universally accepted guidelines (Kim, 2014), due to the paucity of high quality evidence from controlled clinical trials guiding pharmacological management and, expecially, for non neuropathic syndromes despite their high frequency (Hansson, 2004). In neuropathic pain, tricyclic antidepressants (e.g., amytriptiline), serotonin and norepinephrine reuptake inhibitors (e.g., duloxetine) and calcium channel α2δ ligands (e.g., gabapentin or pregabalin) are recommended as first-line agents, but data supporting their use is based on studies in peripheral neuropathic pain, while the evidence in central neuropathic pain is very limited (Mulla et al., 2015). A single study suggested lamotrigine to have a moderate effect on CPSP (Klit et al., 2009). Botulinum toxin injections represent the gold standard for the treatment of post-stroke spasticity and related pain (Hillis, 2020). Given their potential for misuse and other adverse effects (McNicol et al., 2013), opioids stand among the third-line therapy and evidence on their effectiveness for post-stroke pain syndromes is even more limited.

### Aim of the Research

The aim of manuscript is to conduct a systematic review and meta-analysis of evidence on the efficacy of opioid and opioid antagonist medications, important and useful under the recommended conditions (Morrone et al., 2017), for reducing post strokepain and improving pain-related symptoms. Agonists and antagonists at opioid receptors were included in the search. In the brain area subjected to stroke, altered perfusion (Strahlendorf et al., 1980) and changes in opioid neurotransmission (Baskin and Hosobuchi, 1981; Willoch et al., 2004) were suggested to be positively affected by naloxone; incidentally, opioids can reduce blood flow during cerebral ischemia, through inhibition of the release of noradrenaline in the *locus coeruleus* (Budd, 1985). Moreover, naloxone and kappa opioid receptor antagonists were tested in acute ischemic stroke showing in some cases benefit and improvement of neurological conditions (Fallis et al., 1984; Jabaily and Davis, 1984; Perey et al., 1984; Adams et al., 1986; Czlonkowska and Cyrta, 1988; Federico et al., 1991; Czlonkowska et al., 1992; Clark et al., 1996; Lyden, 1996; Clark et al., 2000). Levorphanol, an opioid agonist with high affinity for all the mu, delta and kappa opioid receptors, reported to interact with both N-methyl-D-aspartate (NMDA) receptors and serotonin and norepinephrine uptake (Codd et al., 1995), was included because of its favourable pharmacodynamic and pharmacokinetic characteristics and it showed efficacy in neuropathic pain (Le Rouzic et al., 2019). Oliceridine, a novel mu opioid agonist, was included because it can confer analgesia with less respiratory depression (Dahan et al., 2020).

## Methods

This work was conducted according to the PRISMA (Preferred Reporting Items for Systematic reviews and Meta-Analyses) recommendations (Liberati et al., 2009; Moher et al., 2009).

The systematic review focused on the following question: are opioids effective in reducing pain after stroke and improving pain-related symptoms? Detailed PICOS (i.e., participants, interventions, comparisons, outcomes, study design) framework is shown below:

Participants: patients with pain after stroke; - Intervention: opioid and opioid antagonist medications; - Comparison: placebo or usual/other treatment; - Outcomes: 1) improvement of assessed pain intensity and 2) of pain-related outcomes (e.g., mood, quality of life); - Design of the studies: clinical trials.

The efficacy and safety of opioids on intractable post-stroke pain is a fundamental gap of knowledge due to the lack of studies. Therefore, our systematic review and meta-analysis addresses this broad question, providing an overview of the existing evidence also originating from studies with different design and prompting further future research (Peters et al., 2015). This research aims at highlighting whether opioids and their antagonists are used in post-stroke pain, including medications with different mechanisms of action, and if they are safe and efficacious on the primary outcome of pain reduction and on secondary related outcomes like physical functioning.

### Eligibility Criteria

Studies eligible to be included in this systematic review and meta-analysis were required to meet the following criteria:

clinical trials assessing the effects of opioids on pain in post-stroke patients. No restrictions were placed on the publication date, study duration or follow-up; - patients of any age or ethnicity with post-stroke pain; - interventions include opioids.

Studies meeting the following criteria were excluded from the review:

*in vitro* and *in vivo* animal studies, narrative or systematic reviews and meta-analysis, abstracts and congress communications, proceedings, editorials and book chapters; - clinical trials assessing the effects of other pharmacological treatments (e.g., tricyclic antidepressant, pregabalin) or non - pharmacological management strategies (e.g., neurostimulation techniques); - studies not published in English.

Primary outcomes of interest were changes in objective measures of pain intensity (e.g., pain visual analog scale VAS) and secondary outcomes of interest were changes in pain-related outcomes (e.g., quality of life and physical functioning).

### Search Strategy

The literature search was conducted on PubMed/MEDLINE, Scopus, Web of Science and Cochrane Library databases for peer-reviewed studies on opioid medications for the treatment of post-stroke syndromes and published from databases inception until August 31^st^, 2020 (date of last search). The search strings consisted in a combination of the following keywords: “stroke,” “post-stroke pain,” “pain after stroke,” “central post-stroke pain,” “CPSP,” “shoulder post-stroke,” “thalamic pain syndrome,” “central pain syndrome,” “shoulder hand syndrome,” “complex regional pain”; “Dejerine Roussy,” “facial pain,” “headache,” “facial neuralgia,” “trigeminal autonomic cephalalgia,” “temporomandibular joint disorders,” “allodynia,” “pain secondary to spasticity,” musculoskeletal pain,” “myofascial pain,” “neuropathic pain,” “opioids,” “methadone,” “tramadol,” “codeine,” “morphine,” “buprenorphine,” “oxycodone,” “fentanyl,” “tapentadol,” “loperamide,” “oxymorphone,” “hydrocodone,” “levorphanol,” “sufentanil,” “remifentanil,” “R-dihydroetorphine,” “Morphine-6-glucuronide,” “oliceridine,” “naloxone,” “naltrexone.”

### Study Selection

Two authors independently screened titles and abstracts of the studies in agreement to the previously established inclusion and exclusion criteria. The reference lists of relevant papers were inspected for additional studies potentially missed in the database search. Any disagreement was planned to be solved by consensus or by consulting a third Author.

### Data Collection Procedure

Two authors independently extracted the following data, according to the PICOS framework discussed above: study design, sample size, subtype of post-stroke pain syndrome, interventions, route of drugs administration, comparators, outcomes of interest (primary and secondary), drop-out rates, adverse effects.

### Data Analysis

A systematic and descriptive analysis of the results was provided with information presented in the text and tables. The narrative synthesis has been carried out according to the Cochrane Consumers and Communication Review Group guidelines (Ryan, 2013). Risk of bias and quality of the studies have been assessed, considering study limitations including lack of allocation concealment, lack of blinding, selective outcome reporting bias, inadequate sample or lack of sample size calculation. The revised Cochrane risk of bias tool for randomized trials RoB2 (Sterne et al., 2019) has been used. only the randomized clinical trials included were subjected to meta-analysis to assess imprecision. Indeed, the quality of the body of evidence for both outcomes was rated through the GRADE (Grading of Recommendations, Assessment, Development and Evaluations) system providing the evidence profile including the quality assessment and the summary of findings (Guyatt et al., 2011). Absolute and relative risk with 95% confidence intervals (CI) were calculated using the Cochrane Review Manager 5.3 (RevMan5.3; Copenhagen: The Nordic Cochrane Center, The Cochrane Collaboration). The random effect model (DerSimonian and Kacker, 2007) was used to manage eventual heterogeneity of the studies and to assess intra- and inter-study variation. In particular, for the assessment of inconsistency in results, since the retrieved studies number is small, the Higgins I^2^ value was calculated to assess the heterogeneity of the studies (Higgins and Thompson, 2002). Relative risk below one favors the intervention (opioids) rather than the control/other treatment. Subgroup analysis, sensitivity testing and meta-regression have been performed to evaluate the impact and the causes of heterogeneity and publication bias has been assessed through Egger’s linear regression test to measure funnel plot asymmetry, adjusted through “trim and fill” method (Egger et al., 1997; Duval and Tweedie, 2000; Sterne and Egger, 2001).

## Results

### Identification and Selection of the Studies

**FIGURE 1:**
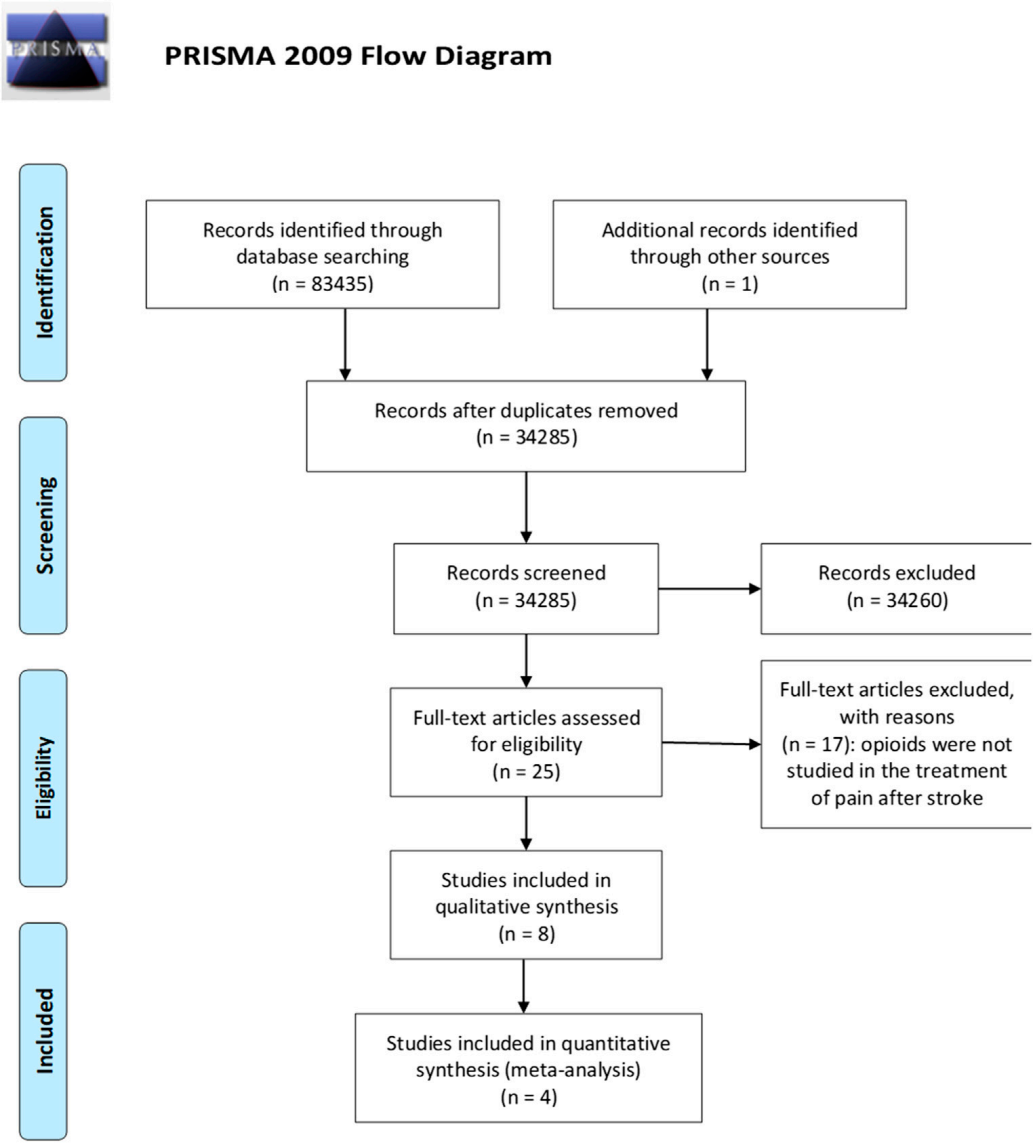
Process of selection of the studies eligible for the qualitative analysis according to the PRISMA guidelines (Moher et al., 2009).

The literature search retrieved a total of 83,435 results. The 83,435 references obtained have been searched for duplicates, leaving 34,285 articles to screen. After titles and abstract screening, not original articles like reviews, book chapters and conference proceedings have been eliminated leaving 24,950 titles and abstracts to screen. After elimination of *in vivo* and *in vitro* studies 2,736 have been screened to exclude observational and retrospective studies, thus leading to 2,531 clinical studies, among which 25 were obtained for full-text reading. One of these trials (Fallis et al., 1984) was not available in full text and one significant paper (Yamamoto et al., 1991) was further identified by the inspection of the reference lists of the relevant records. Eight studies met the inclusion criteria and were therefore included in qualitative synthesis. The four randomized controlled trials (RCTs) (Bainton et al., 1992; Attal et al., 2002; Maier et al., 2002; Rowbotham et al., 2003) were subjected to meta-analysis. The selection process is illustrated in the PRISMA flow diagram (Figure 1).

### Qualitative Summary and Narrative Analysis

The 8 included articles (Budd, 1985; Yamamoto et al., 1991; Bainton et al., 1992; Yamamoto et al., 1997; Attal et al., 2002; Maier et al., 2002; Rowbotham et al., 2003; Saitoh et al., 2003) were clinical trials meeting the previously mentioned inclusion criteria. Studies were grouped according to the intervention (i.e., type of opioid medication), following the Cochrane Consumers and Communication Review Group guidelines (Ryan, 2013). Details of the included studies are summarized in Tables 1 and 2.

**TABLE 1:** Summary of the characteristics of study design of the trials included in qualitative analysis.

Study	Attal et al. (2002)	Bainton et al. (1992)	Budd (1985)	Maier et al. (2002)	Rowbotham et al. (2003)	Yamamoto et al. (1991)	Yamamoto et al. (1997)	Saitoh et al. (2003)
Study design	Randomized, double-blind, placebo-controlled and crossover	Randomized, double-blind, placebo-controlled and crossover	Single-arm trial	Multicenter prospective, randomized, double-blind placebo-controlled and crossover	Randomized, double-blind, dose-response	Single-arm trial: drug challenge test	Single-arm trial: drug challenge test	Single-arm trial: drug challenge test
Patient sample and pain condition	Patients with CPSP (N = 6) or pain due to spinal cord injury (N = 9)	Patients with CPSP (N = 20)	Patients with CPSP (N = 13)	49 patients with neuropathic or nociceptive pain syndromes (CPSP = 2)	81 patients with chronic neuropathic pain of different etiology (CPSP = 10)	Twenty-five patients suffering from intractable deafferentation pain (thalamic/suprathalamic lesions n = 16; brainstem lesions n = 2; spinal cord lesions n = 2; peripheral nerve lesions n = 5)	Thirty-nine patients with intractable hemibody CPSP associated with dysesthesias and allodynia (twenty-five affected by a small thalamic infarct or hemorrhage and fourteen affected by infarct or hemorrhage in the posterior limb of the internal capsule or subcortical parietal area sparing the thalamus)	Nineteen patients with central and peripheral deafferentation pain (seven who had thalamic hemorrhage, one putaminal hemorrhage, one pontine hemorrhage, six brachial plexus injury, two phantom limb pain, one spinal cord injury and one pontine injury)
Intervention	First phase: intravenous infusion of morphine (9–30 mg; mean dosage, 16 mg) for a 20 -minute period; infusion of saline solution was conducted on a separate session after 2 weeks Second phase: within one week after the second infusion, all patients began to take sustained release oral morphine (starting from 20 mg/d during four weeks up to the maximum tolerated dosage)	First intravenous injection of naloxone (8 mg in a 20 ml manufacturer’s vehicle) or of placebo (20 ml of saline solution).The second injection took place 2 or 3 weeks later and contained either naloxone or placebo, depending on previously injected compound	Twenty treatments with intravenous naloxone (from 4.0 to 8.0 mg)	Sustained-release morphine in the first week and placebo in the second week (or reverse order)	Low-strength group: eight weeks levorphanol treatment (max. daily dosage 3.15 mg) High-strength group: eight weeks levorphanol (max. daily dosage 15.75 mg)	Drug challenge tests including morphine test to predict the efficacy of brain stimulation therapy	Drug challenge tests including morphine test to predict the efficacy of brain stimulation therapy	Drug challenge tests including morphine test to predict the efficacy of brain stimulation therapy
Route of drug administration	First phase: intravenous Second phase: oral	Intravenous	Intravenous	Oral	Oral	Intravenous	Intravenous	Intravenous
Comparator	Saline (0.9% NaCl)	Saline (0.9% NaCl)	NA	NS	Low-strength levorphanol	Saline (0.9% NaCl)	Saline (0.9% NaCl)	Saline (0.9% NaCl)

CPSP, Central Post-Stroke Pain; DS, Depression Scale; MPI, Multidimensional Pain Inventory; NA, not applicable; NaCl, sodium chloride; NRS, Numerical Rating Scale; NS, not specified; OAES, Opiate Agonist Effects Scale; OWS, Opiate Withdrawal Scale; PMS, Profile of Mood States; SC-S, Symptom Complaint Score; SDMT, Symbol Digit Modalities Test; VAS, Visual Analogue Scale; VRS, Visual Rating Scale.

**TABLE 2:** Summary of the findings of the studies included in qualitative analysis.

Study	Attal et al. (2002)	Bainton et al. (1992)	Budd (1985)	Maier et al. (2002)	Rowbotham et al. (2003)	Yamamoto et al. (1991)	Yamamoto et al. (1997)	Saitoh et al. (2003)
Primary outcomes	First phase: spontaneous pain = ongoing pain intensity (VAS); evoked pain = intensity of allodynia (VAS); intensity of mechanical pain (VAS); intensity of thermal pain (VAS) Second phase: mean pain intensity (VAS)	Pain intensity (VAS and 5 – word pain score) assessed immediately after the injection	Changes in pain state (direct questioning)	Pain intensity (NRS) Pain tolerability (VRS) Rate and intensity of adverse effects (VRS)	Daily pain intensity (VAS) Pain relief (NRS)	Pain assessed through a visual analog scale	Pain assessed through a visual analog scale	Pain assessed through a visual analog scale and the McGill Pain Questionnaire
Secondary outcomes	Global assessment of pain relief (complete, a lot, moderate, slight, none, or worse pain) Reports of side effects (direct questioning)	Long-term pain intensity reduction (VAS)	Pain relief duration	Sleep quality (VRS) Physical fitness and endurance (NRS) Pain disability index (NRS) Mental state and mood (NRS) Depression (DS) Intensity of symptoms (SC-S)	Mood disturbances (PMS) Quality of life (MPI) Cognitive functioning (SDMT) Symptoms related to agonist and antagonist activity (OAES; OWS) Number of capsules/day Blood levorphanol levels	NA	NA	NA
Results	^a^First phase: morphine significantly reduced dynamic mechanical allodynia (in 9 patients reduction of 50% of pain intensity - VAS) respect to placebo; no significant differences on ongoing pain intensity between morphine and placebo ^a^Second phase: 3 patients out of 15 still took oral morphine after one year follow-up, reporting a 50–70% reduction of mean pain intensity measured with VAS	Inconsistent effects of naloxone compared to placebo on pain intensity reduction: mean ±SE of VAS for naloxone (− 9.35 ± 4.86) vs saline (− 10.05 ± 4.99) Pain relief obtained either with naloxone or placebo was not maintained beyond one day after the injection	7 patients experienced analgesia within 5 min of the completion of naloxone administration lasting from 4 days to 2 and a half years	2 CPSP patients were classified as partial responders (^a^mean pain intensity from 7.8 to 5.6 after morphine; tolerable side effects) ^a^Pain intensity reduction correlated with improvement of physical function ^a^Other secondary outcomes measures did not show significant improvement after morphine treatment compared to placebo	^a^Pain reduction from baseline (high-strength 23 mm vs low-strength 14 mm VAS) ^a^66% patients under high-strength treatment reported pain relief ^a^No significant changes in total mood disturbance in either treatment group ^a^No significant changes in quality of life measures in either treatment group ^a^No significant changes in cognitive functioning in either treatment group ^a^Fewer capsules each day for the high-strength group compared to low-strength (11.9 ± 5.5 vs. 18.3 ± 4.3) ^a^Mean blood levorphanol level closely mirrored the ratio of the actual levels of levorphanol intake in either treatment group	Only 2 patients with thalamic or suprathalamic lesions were responding to morphine	8 patients with CPSP were sensitive to morphine	5 patients resulted responding to morphine
Drop – out rates	^a^First phase: None ^a^Second phase: 60% of patients dropped out because of insufficient pain relief and/or side effects	NA	NA	^a^Only 1 patient dropped the trial	7 out of 10 patients with CPSP dropped	NA	NA	NA
Adverse effects	^a^Nausea, somnolence, headache (mild, rapidly reversible) mainly for morphine administration (60% patients) ^a^Somnolence after placebo (40% patients)	Slight side effects (i.e., rise in pulse rate, sweating, tremor, salivation, pain, nausea, faintness) either after naloxone	Slight transitory changes in heart rate (increase of 10–40 beats/min)	^a^Severe side effects (constipation, vomiting, nausea, sedation and micturition disturbances) occurred in 58% of patients under morphine and in 22% of patients under placebo, independently of dose	^a^Physical or psychological adverse events, treatment failure, lack of adherence	NS	Two patients reported an increase in pain with transient abnormal sensations and anxiety in the ketamine test	NS

CPSP = Central Post-Stroke Pain; DS = Depression Scale; MPI = Multidimensional Pain Inventory; NA = not applicable; NaCl = sodium chloride; NRS = Numerical Rating Scale; NS = not specified; OAES = Opiate Agonist Effects Scale; OWS = Opiate Withdrawal Scale; PMS = Profile of Mood States; SC-S = Symptom Complaint Score; SDMT = Symbol Digit Modalities Test; VAS = Visual Analogue Scale; VRS = Visual Rating Scale.

a Considering the whole sample (no separation between patients with CPSP and those with other types of pain).

### Morphine

Two studies assessed the analgesic effect of morphine on CPSP (Attal et al., 2002; Maier et al., 2002). Attal et al. (2002) performed a double-blind, placebo-controlled, crossover study with the two-fold aim to evaluate the efficacy of intravenous morphine on spontaneous and evoked pain and the long-term benefit of oral morphine on neuropathic pain caused by spinal cord injury or stroke. They reported that the analgesic effect of intravenous morphine regarded only some components of evoked pain (i.e., the intensity of brush-induced allodynia) and that the effects of morphine on ongoing pain were not significantly different from those of the placebo and some patients were reported to receive other pharmacological treatment for pain. Regarding the long-term benefit of oral morphine, only three patients were reported to be still treated after 1 year with persistent pain relief, while the others dropped out before three months due to side effects. It is however unclear whether the patients still on oral morphine treatment at 1 year were those belonging to the group of neuropathic pain caused by stroke or by spinal cord injury. Maier et al. (2002) conducted a prospective, randomized, double-blind, placebo-controlled, crossover study on forty-nine patients with either neuropathic (of which only two had CPSP) or nociceptive pain syndromes, assessing the efficacy and effectiveness of 1 week of oral morphine administration. In fact, the MONTAS study assessed the efficacy of morphine on chronic non-tumor associated pain syndromes (Maier et al., 2002). An interdisciplinary consensus protocol on compulsory and optional treatments for pain, excluding strong opioids was followed before inclusion. The two patients with CPSP were classified as partial responders according to the reduction from 7.8 to 5.6 of mean pain intensity measured with an 11 points Numerical Rating Scale (NRS) and to the overall tolerability of adverse effects connected with opioid medications. Pain reduction was reported to correlate with improvement of physical function. Moreover, the Authors found a reduction of pain disability, depression score, mood and exercise endurance, secondary pain-related outcomes. The pharmacological background of intractable CPSP was characterized in three studies through morphine tests: two by Yamamoto and coworkers (Yamamoto et al., 1991; Yamamoto et al., 1997) and one by Saitoh and collaborators (Saitoh et al., 2003). The first study evaluated deafferentation pain using the morphine/thiamylal test enrolling twenty-five patients suffering from intractable deafferentation pain (thalamic/suprathalamic lesions n = 16; brainstem lesions n = 2; spinal cord lesions n = 2; peripheral nerve lesions n = 5) (Yamamoto et al., 1991). The morphine test consists in intravenously administering 3 mg morphine hydrochloride every 5 min up to reach 18 mg, followed by injection of naloxone to reverse thus confirming the effect of morphine, and assessing pain through a visual analog scale at 5 min intervals (Yamamoto et al., 1991). All the patients included were resistant to pharmacological therapy. According to the results, only two patients with thalamic or suprathalamic lesions were responding to morphine and thiamylal (Yamamoto et al., 1991). In the second study by Yamamoto et al. (1997) thirty-nine patients with intractable hemibody CPSP associated with dysesthesias and allodynia (twenty-five affected by a small thalamic infarct or hemorrhage and fourteen affected by infarct or hemorrhage in the posterior limb of the internal capsule or subcortical parietal area sparing the thalamus) were subjected to the morphine and thiamylal tests and only twenty-three recent cases were subjected to the ketamine test. All the patients had been received treatment with tricyclic and heterocyclic antidepressants, benzodiazepines and non-narcotic analgesics without satisfactory pain reduction. During this study, eight patients with CPSP were sensitive to morphine experiencing transient satisfactory pain reduction (Yamamoto et al., 1997). In the study by Saitoh and colleagues (Saitoh et al., 2003) nineteen patients with central and peripheral deafferentation pain (seven who had thalamic hemorrhage, one putaminal hemorrhage, one pontine hemorrhage, six brachial plexus injury, two phantom limb pain, one spinal cord injury and one pontine injury) of which eighteen underwent drug challenge test and pain was assessed through a visual analog scale and the McGill Pain Questionnaire. All the patients were treated with various medications including NSAIDs, anticonvulsants and antidepressants also used in combination, without sufficient reduction of pain. Among these patients, five were sensitive to morphine (Saitoh et al., 2003).

### Levorphanol

One study (Rowbotham et al., 2003) evaluated the efficacy of low and high doses of the opioid agonist levorphanol on eighty-one patients with neuropathic pain of different aetiology (patients with CPSP were ten). All patients had not achieved pain relief with previous non opioid medications and a trend towards previous use of low dose opioid was reported in the low-strength group. Compared to low ones, high doses of levorphanol resulted in higher rates of reduction in the intensity of neuropathic pain, considering the whole patient sample; however, high doses of levorphanol also resulted in more severe side effects that led to higher drop-out rates. Despite the additional outcomes of affective distress and interference with functioning were reduced, no difference between groups were observed. Moreover, pain relief was less frequent for patients suffering from CPSP. Pain effect on physical functioning was evaluated only in this study (Rowbotham et al., 2003).

### Naloxone

Two studies assessed the effect of the opioid antagonist naloxone on CPSP (Budd, 1985; Bainton et al., 1992). Budd (1985) performed a single group study on thirteen patients with pain due to thalamic syndrome resistant to prior analgesic or other therapies and reported analgesia, assessed by direct questioning, for seven patients after twenty intravenous administrations of naloxone. The duration of the effects varied from 4 days to two and a half years. On the other hand, the placebo-controlled study by Bainton et al. (1992) failed to demonstrate the efficacy of intravenous administration of naloxone in alleviating CPSP.

### Risk of Bias Assessment

**FIGURE 2:**
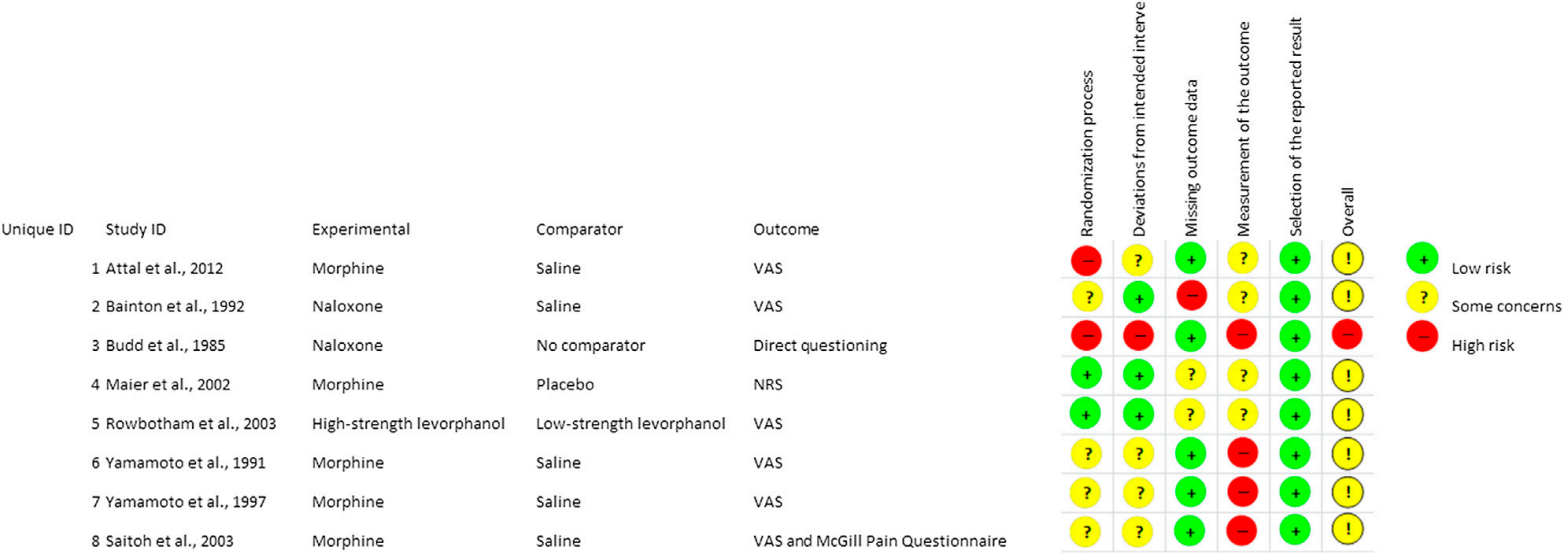
Summary of risk of bias assessment for the studies included in qualitative analysis from low to some concerns and high. The mark (+) indicates low risk of bias and the mark (−) indicates high risk of bias.

Four of the studies are randomized clinical trials (Bainton et al., 1992; Attal et al., 2002; Maier et al., 2002; Rowbotham et al., 2003), one is a single arm trial (Budd, 1985) and three (Yamamoto et al., 1991; Yamamoto et al., 1997; Saitoh et al., 2003) are drug challenge tests. Therefore, the included studies are very heterogeneous in terms of study design. Moreover, four studies (Budd, 1985; Yamamoto et al., 1991; Yamamoto et al., 1997; Saitoh et al., 2003) included one single group without control. In the study by Rowbotham et al. (2003) the groups compared are high and low strength. The lack of a control arm can rise some concerns in terms of bias as for concealment. The population enrolled is heterogeneous across the eight studies and the number of patients is small for all the trials except for Maier et al. (2002) and for Rowbotham et al. (2003); however, the CPSP patients are only two and ten, respectively. Moreover, in the MONTAS study, with crossover design, number needed to treat and number needed to harm are reported to have been calculated only with reference to first week since the results of the second week could feel the effect of opioid withdrawal symptoms. Compliance to treatment has been assessed by pill counts and repeated urine screening, revealing only minor protocol violations. Interestingly, double masking was applied to three trials (Bainton et al., 1992; Attal et al., 2002; Rowbotham et al., 2003), but for the study by Attal et al. (2002) it was reported that seven patients and the examiner (in ten cases) had identified the active treatment, thus impairing blindness. In the study of Maier et al. (2002), a random generator was used for patients randomization and the medication package was blinded. The summary of risk of bias assessment according to intention-to-treat analysis is reported in Figure 2.

### Meta-Analysis and GRADE Evidence Profile (EP)

**FIGURE 3:**
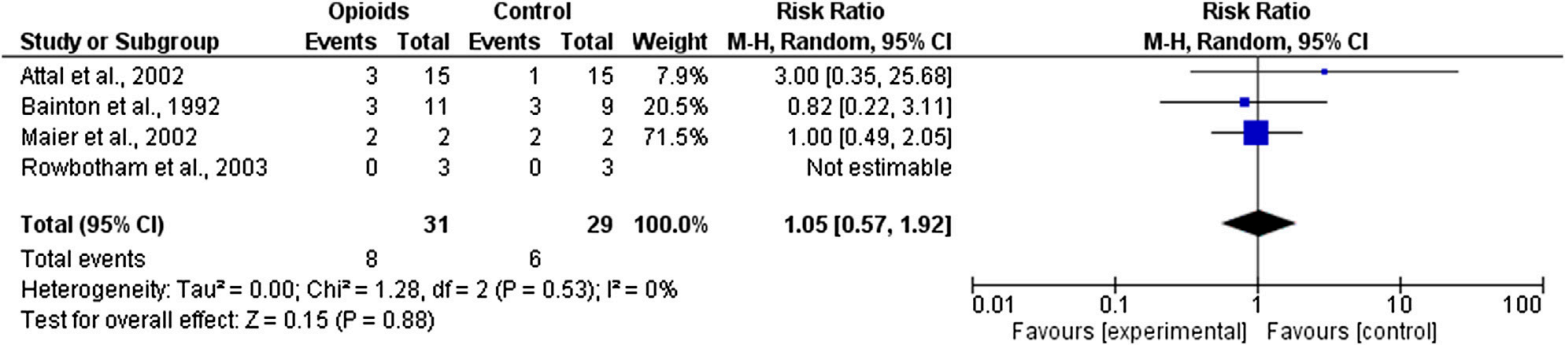
Forest plot for efficacy of opioid agonists and antagonists in pain reduction in trials included in the meta-analysis.

**FIGURE 4:**
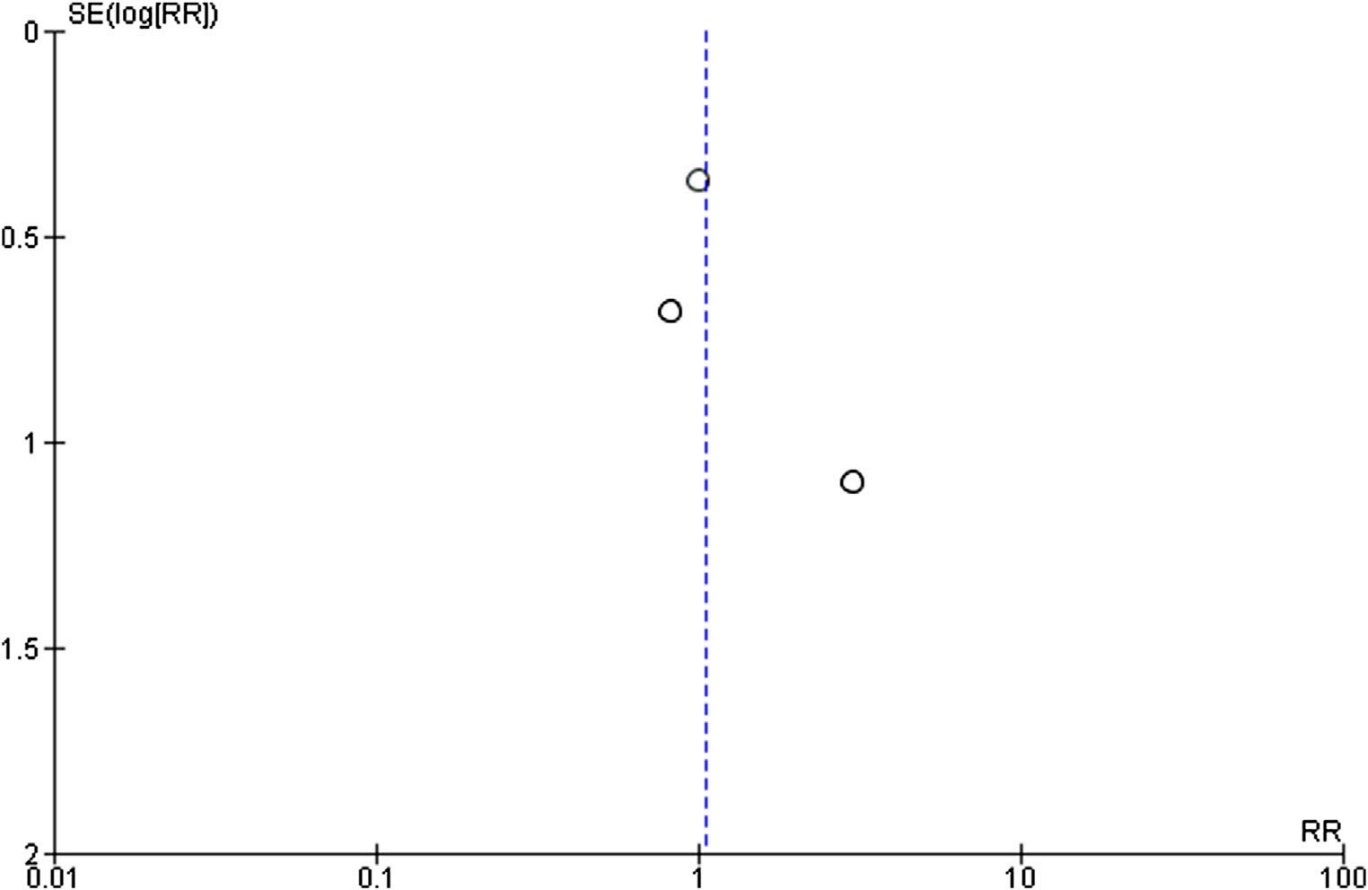
Funnel plot for efficacy of opioid agonists and antagonists in pain reduction in trials included in the meta-analysis.

The quality of evidence of the two selected outcomes, i.e. analgesic efficacy of opioids in post-stroke pain and effectiveness on pain-related domains, was rated through the GRADE system (Guyatt et al., 2008; Guyatt et al., 2011). The quality assessment was based on: Limitations; Inconsistency; Indirectness; Imprecision and Publication bias. For each outcome, the four retrieved randomized clinical trials (Bainton et al., 1992; Attal et al., 2002; Maier et al., 2002; Rowbotham et al., 2003) were subjected to meta-analysis (Figure 3), for the assessment of absolute and relative risk and width in the CIs to calculate imprecision, with funnel plot for the evaluation of publication bias (Figure 4). The GRADE assessment reveals very low quality of evidence for the outcome of pain reduction and low quality of evidence for pain-related outcomes. This meta-analysis follows the Initiative on Methods, Measurement, and Pain Assessment in Clinical Trials (IMMPACT) recommendations (Turk et al., 2003). The core outcome domains for clinical trials of chronic pain treatment efficacy and effectiveness have been identified as pain; physical functioning; emotional functioning; participant ratings of global improvement; symptoms and adverse events; participant disposition (including adherence to the treatment regimen and reasons for premature withdrawal from the trial) (Turk et al., 2003). A meaningful decrease in chronic pain representing a clinically important difference in pain intensity is determined as change of approximately 2.0 points of Numerical Rating Scale (NRS) or 30–36% (Dworkin et al., 2008). Therefore, administration of opioids (agonists or antagonists) is not associated to meaningful pain relief (Relative Risk RR 1.05; 95% CI 0.57–1.92; I^2^ = 0%; *p* = 0.53; Figure 3) and data are influenced by the paucity and the design of the studies. Though in agreement with I^2^, heterogeneity allows comparison of these RCTs, RR is not estimable for the study by Rowbotham et al. (2003), since there is not a real control arm, but a high- and a low-strength arm. This occurs also for the pain-related outcome, thus influencing the RR calculation (RR 1.00; 95% CI 0.49–2.05; heterogeneity not applicable; Figure 4), since only two RCTs evaluate this outcome. In this study (Rowbotham et al., 2003) no significant changes in total mood disturbance and in quality of life were reported in either treatment group. In the study by Maier et al. (2002), the improvement of pain-related outcomes exerted by morphine administration reached statistical significance (*p* ≤ 0.05) only for pain disability and sleep quality. Therefore, RR is based only on the study by Maier et al. (2002), for pain-related outcome, thus forest and funnel plots are not reported; evidence coming from a single trial is uncertain. According to the forest plot in Figure 3 the results do not favor the experimental treatment (opioid agonist or antagonist) rather than the placebo for the outcome of pain reduction.

The GRADE assessment is based on rating of the following four domains:

1. Limitations: lack of allocation concealment and/or of blinding, loss to follow-up, failure to adhere to an intention to treat analysis and failure to report outcomes. This key outcome was downgraded for failure of concealment and blinding (Attal et al., 2002), lack of control arm and large loss to follow-up (Rowbotham et al., 2003) and minor protocol violations (Maier et al., 2002) for pain reduction; for the same reasons, this outcome was downgraded for pain-related outcomes.
2. Inconsistency: variability in results across studies can be due to true differences in treatment effect. The rate of this domain was downgraded since efficacy is partial across the studies for pain reduction, and for pain-related outcomes since results are opposite in the two RCTs.
3. Indirectness: differences between the population, intervention, comparator and outcome of interest and those included in the relevant studies. This key outcome was downgraded both for pain reduction and for pain-related outcomes since these studies are conceived for populations including mixed types of pain and not only post-stroke pain as defined in the PICOS.
4. Imprecision: wide CIs. The retrieved RCTs include relatively few patients and thus have wide CIs.
5. Publication bias: studies showing no significant results are often unpublished. Due to the asymmetry of the funnel plot and to the paucity of studies, there is strong suspicion of publication bias. Therefore, the rating of this domain is defined as “likely”.

The EP with quality assessment and Summary of Findings (SoF) is reported in Table 3.

**Table 3:** GRADE rating of overall quality of evidence: quality assessment and summary of findings of the body of evidence for pain reduction and pain-related outcomes.

Quality assessment						Summary of Findings		
No of studies (Design)	Study limitations	Inconsistency	Indirectness	Imprecision	Publication bias	Relative effect (95% CI) ^b^	Risk difference (95% CI)	Quality
Analgesic efficacy^a^: 4 (RCT)	Serious limitations (−1)	Serious inconsistency (−1)	Serious indirectness (−1)	Imprecision^c^	Likely	RR 1.05 (0.57–1.92)	RD 0.06 (−0.12 to 0.24)	Very low ○○○⊕
Effectiveness on pain-related domains^a^: 2 (RCT)	Serious limitations (−1)	Serious inconsistency (−1)	Serious indirectness (−1)	Imprecision^c^	Likely	RR 1.00 (0.49–2.05)	RD 0.00 (−0.60 to 0.60)	Very low ○○○⊕

RCT, Randomized clinical trial; CI, Confidence Interval.

a Please refer to PICOS outcomes in the Methods paragraph.

b Relative risks (95% CI) are based on random effect models.

c The retrieved RCTs include relatively few patients.

## Discussion

Post-stroke pain is a complex condition representing both an underdiagnosed and an undertreated chronic consequence of cerebrovascular events. Pain after stroke encompasses neuropathic and nociceptive features and can be either spontaneous or evoked, constant or intermittent (Klit et al., 2009). It consists in a variety of pain syndromes going from CPSP, CRPS, pain secondary to spasticity and musculoskeletal pain, which may occur with variable prevalence and that can be present simultaneously being different and difficult to characterize and adequately treated in each individual patient (Delpont et al., 2018). Post-stroke pain management is complex, considering its multifaceted nature and the existence of multiple pharmacological and non-pharmacological therapeutic approaches depending on the pain subtype. In fact, according to the underlying pathophysiology a different management is needed (Harrison and Field, 2015): local neuromuscular blockade for pain secondary to spasticity, mechanical stabilization and rehabilitation with shoulder strapping in musculoskeletal pain, while first line for neuropathic pain consists in α2δ-1 ligands and antidepressants and in case of severe resistant pain opioids can represent an adjuvant treatment. Data and evidence for second and third-line therapies are even more scant. Also opioid antagonists could exert some efficacy in pain after stroke since anomalous perfusion (Strahlendorf et al., 1980) or increased levels of endogenous opioids (Baskin and Hosobuchi, 1981; Willoch et al., 2004) can affect the area subjected to stroke. Indeed, naloxone is studied in acute ischemic stroke. Other opioids exert peculiar actions like levorphanol that can interact with both NMDA receptors and serotonin and norepinephrine uptake (Codd et al., 1995). This systematic review and meta-analysis evaluated clinical trials investigating the effect of opioids and opioid antagonists on pain after stroke and its functional consequences. A small number of studies (i.e., 8 results) met the inclusion criteria and was therefore included in the qualitative analysis and narrative synthesis (Budd, 1985; Yamamoto et al., 1991; Bainton et al., 1992; Yamamoto et al., 1997; Attal et al., 2002; Maier et al., 2002; Rowbotham et al., 2003; Saitoh et al., 2003). Despite the differences among study design (i.e., placebo-controlled, single arm, dose-response) and mechanism of the various opioids investigated (i.e., morphine, naloxone, levorphanol), nearly all the included studies (except one) showed an overall only slight analgesic effect of opioid medications on CPSP, with various primary outcomes (VAS/NRS, pain relief, pain tolerability) and less consistent effects on other pain-related symptoms. All the patients included in these studies suffered from intractable pain resistant to previous analgesic treatment. Because of the small number of studies and patients, and the different study design across them, no robust evidence can be drawn (Sharpe, 1997). Due to their design, the three drug challenge tests and the study of Budd (1985) are single group without control and lacking information concerned with masking of patients, deliverers and assessors, thus rising some concern in terms of concealment. Double masking was applied to three trials (Bainton et al., 1992; Attal et al., 2002; Rowbotham et al., 2003) and, in the MONTAS study, patients were randomized using a random generator and received the same blinded medication package. However, in the study by Attal et al. (2002), blindness was put at risk since seven patients and the examiner (in ten cases) identified the active treatment. Moreover, studies differed in terms of pain scales used and they are very heterogeneous in terms of population enrolled, impairing directness. Trials are adequately designed but not specifically for post-stroke pain, e.g. the number of patients is small for all the trials except for the MONTAS study and the study by Rowbotham et al. (2003), whereas the CPSP patients are only two and ten, respectively. Moreover, sample power calculation is not reported. Overall, in this population, any estimate of effect for the first PICOS outcome is very uncertain and results are inconclusive due to the small number of studies and of patients: in fact, each of the four important GRADE criteria ranges from moderate/low to very low quality of evidence, downgrading to very low the overall quality of evidence of efficacy of opioids (Atkins et al., 2004; Guyatt et al., 2008; Guyatt et al., 2011). The IMMPACT recommendations support the importance of physical functioning as core outcome for pain (Turk et al., 2003), an issue of the utmost importance in these patients. However, only the study by Rowbotham et al. (2003) included physical functioning as an outcome and the MONTAS study highlighted an improvement of pain-associated sensory and affective variables and disability. Therefore, there is low quality of evidence for the second PICOS outcome.

The poor/unclear response of CPSP to opioids is in keeping with reduced binding to opioids in pain circuitry in CPSP (Willoch et al., 2004). Indeed, CPSP patients show decreased brain opioid receptors binding in posterior midbrain, medial thalamus and the insular, temporal and prefrontal cortices contralateral to pain, being this pattern different from the opioid receptors binding occurring in peripheral neuropathic pain, thus supporting different response of central vs. peripheral neuropathic pain to opioids (Maarrawi et al., 2007). Of interest, despite the wide search criteria we used, all the included studies pertained CPSP, and we found no evidence on other types of post-stroke pain. Indeed, all the included studies were quite old and dating prior to the 2009 CPSP redefinition (Klit et al., 2009), which made the differential diagnosis between CPSP and other types of post-stroke pain clearer and more reliable. Pain assessment represents an important issue in non-communicative patients, who can have difficulties to describe their pain, contributing to behavioral disturbances in some neurological conditions (Scuteri et al., 2017; Scuteri et al., 2018; Scuteri et al., 2019a; Scuteri et al., 2019b). Indeed, in the real-life clinical setting, the use of opioids in patients with post-stroke pain, who are not able to communicate is frequent, but the response to treatment is unclear (Schuster et al., 2020). Post-stroke pain can occur also in patients with neurodegenerative disorders (Scherder and Plooij, 2012) and clinical trials to assess the efficacy and safety of opioids are needed, being the treatment of pain often inappropriate in this population (Scuteri et al., 2017; Scuteri et al., 2018; Scuteri et al., 2020a; Scuteri et al., 2020b). Future double-blind randomized clinical trials designed specifically for post-stroke pain, methodology and statistical power are needed to assess the efficacy and safety of opioids in post-stroke pain and to understand the impact of pain treatment on physical function. In fact, being post-stroke pain often severe, it may be resistant to first line treatments, as it occurs in all of the studies included in the analysis; the latter condition makes treatment with opioids sometimes necessary. In these eight studies morphine induced nausea, somnolence, headache and severe side effects (in 58% of patients of the study by Maier et al. (2002), consisting in constipation, vomiting, nausea, sedation and micturition disturbances), also psychological effects were reported with levorphanol and naloxone caused slight increase in pulse rate, sweating, tremor, salivation, pain, nausea and faintness. In the light of the most rigorous analysis of the literature, it is conceivable that opioid use within a time frame of no longer than 12 weeks is not linked to respiratory depression and to the potential for abuse with overdose death (Dowell et al., 2016). However, further evidence is necessary for the best clinical use of these effective analgesics, also limiting the most serious consequences of inappropriate opioids prescription.

## Data Availability Statement

The original contributions presented in the study are included in the article.

## Author Contributions

DS, GB and PT conceived the study. All Authors have participated in the manuscript preparation and have read and approved the final manuscript.

## Conflict of Interest

The authors declare that the research was conducted in the absence of any commercial or financial relationships that could be construed as a potential conflict of interest.
